# The Role of Keratin in the Mechanisms of Kidney Diseases

**DOI:** 10.1155/bmri/5534800

**Published:** 2026-07-31

**Authors:** Shitian Che, Xiaolu Huang, Shuxiang Yan, Baiyu Feng, Lijun Yin, Anqun Chen

**Affiliations:** ^1^ Department of Nephrology, Hunan Key Laboratory of Kidney Disease and Blood Purification, Institute of Nephrology, The Second Xiangya Hospital at Central South University, Changsha, China; ^2^ National Clinical Research Center for Metabolic Diseases, The Second Xiangya Hospital of Central South University, Changsha, Hunan, China, csu.edu.cn; ^3^ Furong Laboratory, Changsha, Hunan, China

**Keywords:** AKI, cell death, chronic kidney diseases, keratin, kidney diseases

## Abstract

Kidney disease remains a leading cause of global mortality, with its prevalence continuing to rise. Despite advances in understanding glomerular pathologies and acute kidney injury, the progression to irreversible renal failure and its associated systemic complications represent a major unresolved challenge. Keratin, a cytoskeletal protein encoded by 54 genes, is now recognized as a pivotal regulator of renal pathophysiology. Emerging evidence implicates keratin dysregulation in renal pathophysiology through dual mechanisms: maintaining podocyte cytoarchitecture via cytoskeletal stabilization and driving fibrosis through aberrant stress‐response signaling. These processes position keratin as a molecular mediator bridging structural homeostasis and pathological remodeling in kidney diseases. Current research focuses on its diagnostic potential and therapeutic targeting in chronic kidney disease progression. This review synthesizes keratin’s multifaceted roles, highlighting its emerging significance in both glomerular and tubulointerstitial pathologies.

## 1. Materials and Methods

A comprehensive literature search was conducted to identify relevant studies on the role of keratin in kidney diseases. Three major electronic databases, namely PubMed, Scopus, and Web of Science, were systematically searched. The search was performed independently by two reviewers to ensure completeness and minimize potential selection bias.

The following search terms and keywords were used in various combinations, employing Boolean operators (AND, OR) to refine the search strategy: “keratin”, “cytokeratin”, “intermediate filament”, “kidney disease”, “renal disease”, “chronic kidney disease”, “CKD”, “acute kidney injury”, “AKI”, “renal fibrosis”, “tubular injury”, “glomerular disease”, “biomarker”, “prognosis”, “diagnosis”, “therapeutic target”, “pathogenesis”, “mechanism”, “expression”, “upregulation”, “downregulation”, “tubular epithelial cell”, “podocyte”, “animal model”, “cell line”, and “clinical study”. Representative search strings used in PubMed included the following: (“keratin”[Title/Abstract] OR “cytokeratin”[Title/Abstract]) AND (“kidney disease”[Title/Abstract] OR “renal disease”[Title/Abstract] OR “CKD”[Title/Abstract] OR “AKI”[Title/Abstract]) AND (“biomarker”[Title/Abstract] OR “mechanism”[Title/Abstract] OR “pathogenesis”[Title/Abstract]).

The inclusion criteria were as follows: (1) peer‐reviewed articles published in English; (2) studies investigating keratin expression, function, or clinical relevance in kidney diseases; (3) original research articles, reviews, and meta‐analyses. The exclusion criteria were the following: (1) articles not published in English; (2) studies focusing solely on non‐renal tissues without kidney disease context; (3) conference abstracts, editorials, or letters without sufficient data; (4) duplicate publications.

To ensure the inclusion of recent advances in this rapidly evolving field, the search was primarily restricted to publications from the last 5–10 years (approximately 2016–2026), with seminal earlier studies included selectively when historically significant.

The initial database search yielded a total of relevant records. After removing duplicates, the remaining unique records underwent title and abstract screening. Full‐text assessment was subsequently performed, and eligible studies were included for qualitative synthesis in this review. All disagreements between the two reviewers regarding study eligibility were resolved through discussion and consensus.

## 2. Introduction

Keratins, the largest subgroup of intermediate filament (IF) proteins, are broadly classified into acidic Type I (K9–K28, K31–K40) and basic/neutral Type II (K1–K8, K71–K86) isoforms1. In terms of molecular structure, keratin can be further divided into *α*‐keratin and *β*‐keratin. The IF secondary structures of *α*‐keratin and *β*‐keratin are *α*‐helix and *β*‐fold, respectively, which is also the main difference between the two [1].

**Figure 1:**
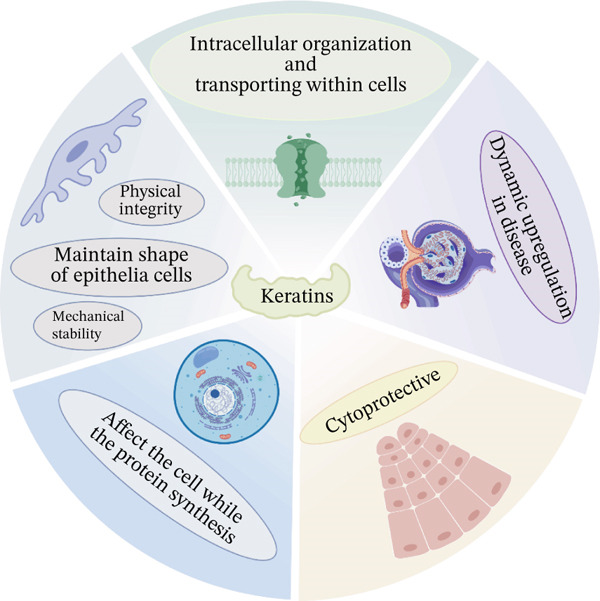
The main function of keratin. This figure was originally created by the authors from self‐photographed and self‐drawn elements; no previously published material was used.

The complex structure of keratin also determines its diverse functions. The main functions of keratins are as follows (Figure 1): (1) Preserving the physical soundness, mechanical steadiness, and form of epithelial cells; (2) Intracellular organization and transport within cells; (3) Keratins are considered cytoprotective; (4) A undergoing dynamic upregulation in disease states; (5) The cell’s migration, growth, and proliferation are impacted by protein synthesis [2].

What about keratin in the kidney? Different types of keratins are distributed differently in the kidney and play different roles in physiology and pathology. Currently, not all types of keratins have been found in the kidney, and the more studied ones are K8, K18, K7, and K19. K8/K18, a regulatory factor in our kidneys [3], is indispensable for sustaining the soundness and mechanical stability of renal tubular epithelial cells. It can affect cell growth and demise through dynamic interactions with nonstructural proteins [4]. Both K7 and K19 are found specifically in parietal epithelial cells, the thin limbs of Henle, and the collecting ducts [5]. As stress‐response proteins, K7/K19 are upregulated during renal epithelial injury and subsequent repair, contributing to enhanced epithelial cell adaptability [6]. Apart from the commonalities with other renal keratins, K8 and K19 have their unique role: they can regulate the stretching response of keratin in epithelial cells, while the phosphorylation of K8 induces the recombination of keratin filaments and promotes the migration of epithelial tumor cells [5]. Another one is K40, which is widely distributed in the kidneys, and more abundant in distal tubular segments. The expression of the type I keratin K40 affects recombinant uromodulin (UMOD) processing and excreting in thick ascending limb (TAL) cells [2].

At present, increasing attention is being given to the role of keratin in kidney disease. Therefore, in this article, we review the distribution, function, and mechanism of keratins in kidney diseases, including acute kidney injury (AKI), renal cancer, and other chronic kidney diseases. Finally, we discuss the pathways by which keratins regulate kidney disease. This review aims to deepen the systematic understanding of keratin in kidney diseases and provide a reference for finding potential targets.

## 3. Role of Keratin in Kidney Disease

The relationship between keratin and various kidney diseases will be discussed in detail in the following sections. As mentioned above, keratin is closely associated with kidney disease. The expression content (or expression site) of some keratin changes during the pathogenesis of kidney disease. The degree of hydronephrosis is linked to K18 expression, and end‐stage renal disease (ESRD)/advanced chronic renal disease (ACRD) stress induces the expression of K7 and K19. Additionally, some keratin plays a critical role in the development of kidney disease, such as K8’s part in RCC.

### 3.1. Role of Keratin in the Pathogenesis of AKI

#### 3.1.1. Keratin Changes in AKI

Previous research has indicated a strong correlation between keratin and acute kidney disease. In a study using a rat model of acute tubular injury induced by mercury chloride poisoning, co‐expression of vimentin and keratin was observed in tubular regeneration. However, normal human tubular epithelial cells only expressed keratin, not vimentin [7].

AKI causes extensive keratin signaling in injured kidneys, but not all subtypes of keratin are associated with AKI. In AKI, the expression of K8, as a key injury response factor, was elevated [8]. Chromosome in situ hybridization (CISH) and immunofluorescence showed that K8 transcription was also elevated and accompanied by increased protein. Moreover, K18 expression is significantly elevated in renal ischemia–reperfusion injury (IRI), and erythropoietin (EPO) can amplify its expression [9]. In addition, an intriguing experiment revealed that in a mouse model with human angiotensin‐converting enzyme 2 (ACE2) expression controlled by a human K18 promoter, intranasal inoculation of high doses of severe acute respiratory syndrome coronavirus 2 (SARS‐CoV‐2) resulted in severe kidney damage [10]. In animal experiments of renal IRI, compared with male mice, the expression of K18, a gene associated with proximal tubule injury, was less increased after ischemia in female mice, and K20 was not upregulated [11]. Studies from our team and others have demonstrated that K20 expression was significantly upregulated in the renal tissue of male AKI mice [12, 13], and that urinary K20 levels was a predictor for CKD following AKI [13–15].

In certain types of acute nephropathy, keratin expression is also affected. For example, in hemolytic AKI, cytokeratin levels rise [16]. Regarding acute rejection in kidney transplantation, K1 may play a role in the pathogenesis [17]. In Donation after Cardiac Death (DCD) kidneys, an 18‐fold increase in renal production of K18 indicates extensive necrotic cell death [18]. Keratin is also expressed positively in atherosclerotic renal vascular hypertension caused by aortic intimal sarcoma [19]. Studies on crescent bodies and keratin indicate that cytokeratin positivity is important for crescent localization and cell origin identification [20, 21].

#### 3.1.2. Mechanism Study of Acute Nephropathy and Keratin

Although the specific mechanism of keratin in acute kidney disease is unclear, relevant studies have shown that keratin plays an important role in certain pathways.

Keratin‐1 may be related to the pathogenesis of acute rejection of renal transplantation [17]. Studies have shown that K1 plays a role in the lectin complement pathway, which is triggered by oxidative stress in endothelial cells. If the recipient has antibodies for endothelial cells, K1 antibodies may be considered autoantibodies. The presence of K1 antibodies is significantly linked to a decline in renal allograft function.

**Figure 2:**
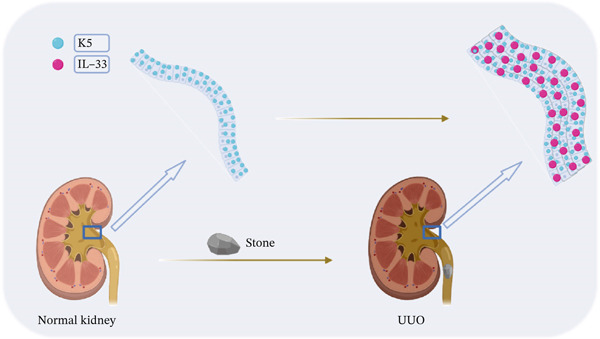
Changes of K5 in UUO‐induced AKI.

In AKI caused by unilateral ureteral blockage (UUO), the IL‐33/ST2 axis mediates activation of the innate immune response and promotes urothelial hyperplasia by modulating urothelial differentiation in obstructive kidney injury [22]. In a normal kidney, the ureteropelvic junction (UPJ) urothelium typically comprises a single layer of urothelial cells expressing keratin 5 (K5). The urothelium at the UPJ in the UUO kidney was significantly thickened, with an increased number of basal urothelium marked by K5+. However, the K5+ urothelium in the fornical remained a single layer. Of note, IL‐33 expression was only detected in the UPJ and upper urinary tract, and it was co‐localized with multiple layers of K5+ basal cells, but not with the single‐layer K5+ medullary urothelial cells in the fornix (Figure 2).

#### 3.1.3. Keratin as a Biomarker of AKI

For many in acute kidney disease, keratin can be a noninvasive biomarker. First, regarding keratin 18, it has been proven to be a nonspecific marker of liver damage, so its value in kidney disease is worth investigating [23]. Most studies support keratin 18 as a marker of renal tubule injury [18], but some do not support the role of keratin 18 epitope M30 in the development of AKI [24]. K1 may serve as a serum biomarker for acute renal transplant rejection, and the HLA‐DR/cytokeratin ratio is also useful in diagnosing rejection [25]. Moreover, keratin positivity can be used as an auxiliary diagnostic indicator in atherosclerotic renal vascular hypertension, hemolytic AKI [6], and osmotic nephropathy [26].

### 3.2. Involvement of Keratin in Chronic Kidney Disease

This section delves into the connection between chronic kidney disease and keratin, dividing it into hereditary and nonhereditary components. In the nonhereditary section, glomerular diseases and renal interstitial diseases will be introduced (Table 1).

**Table 1:** Localization of keratin in kidneys of different species.

IF type	Primary cell type	Protein		Gene	Mol Wt (KDa)	Distribution in healthy human kidney	Distribution in healthy mice
I	Epithelial cells	Keratin	K18	KRT18	40–57	All epithelial cells of the nephron	Only in the collecting duct
			K19	KRT19		The parietal epithelial cells, the thin limbs of Henle, and the collecting ducts	The same as human
II			K7	KRT7	50–70	The parietal epithelial cells, the thin limbs of Henle, and the collecting ducts	The same as human
			K8	KRT8		All epithelial cells of the nephron	Only in the collecting duct

#### 3.2.1. Chronic Glomerulonephritis (CGN)

CGN is the most prevalent form of this kidney disease, marking the final stage following the development of different types of glomerulonephritis. This aggressive renal condition is known for its nephrotic proteinuria, severe renal dysfunction, slow progression, resistance to treatment, and rapid advancement to end‐stage renal failure [27]. Patients often die from uremia and hypertension causing heart failure or cerebral hemorrhage.

According to recent research, keratin expression clearly differs across CGN subtypes:

ANCA‐associated vasculitis (AAV): glomerular K7, K8, K18, and K19 are > 20‐fold higher than healthy controls, with K18 exceeding IgA nephropathy levels; tubulointerstitial K19 rises four‐fold and correlates with crescent count [28].

IgA nephropathy (IgAN): glomerular K18 is > 10‐fold up, strongest in crescentic areas; tubulointerstitial K19 increases ~3‐fold and links to interstitial inflammation [28]. Comparative analysis of differential proteins identified in urine and glomeruli revealed that changes in K10, K1, and K5 are synchronized with the degree of mesangial proliferation observed on renal biopsy and occur earlier than the rise in serum creatinine. This indicates that keratin fragments can reflect active mesangial lesions and are suitable for monitoring disease activity in early‐stage IgA nephropathy [29].

Podocytopathies (MCD/FSGS): no significant keratin up‐regulation, indicating that changes relate to inflammatory proliferation (crescents, mesangial hyperplasia) rather than pure proteinuria. The degree of interstitial fibrosis or tubular atrophy does not significantly influence levels of keratins K7, K8, K18, and K19, indicating that these keratins primarily reflect active injury rather than chronic scarring [28].

#### 3.2.2. Renal Interstitial Diseases

Studies have identified keratin K20 as the starting gun for renal tubular epithelial stress. Upon stress, K20 binds to the nuclear protein PRDX2, suppresses ferroptosis and reduces tubular‐cell loss; when K20 is knocked out, ferroptosis is exacerbated and the area of interstitial inflammation expands 1.8‐fold. Within 30 min of onset, K20 mRNA and protein in tubular epithelia rise markedly, while urinary K20 becomes detectable 2 h after IRI, remains elevated for 14 days and rises far earlier than serum creatinine, indicating that K20 can serve as a noninvasive early‐warning molecule for the onset stage of chronic renal interstitial disease [12, 15, 30].

#### 3.2.3. Hereditary Chronic Kidney Disease

This review will specifically address polycystic kidney disease in the context of inherited chronic kidney conditions. Polycystic kidney disease encompasses both autosomal dominant polycystic kidney disease (ADPKD) and autosomal recessive polycystic kidney disease (ARPKD). The etiology could be due to genetic factors.

1. Tubular cell proliferation, cell transformation into a pre‐tumor state
2. The production of a defective tubular basement membrane (BM) by tubular cells leads to an alteration in BM‐cell interactions [31].

Both ARPKD and ADPKD belong to DPM (ductal plate malformation). The cytokeratin immunophenotype of ductal plate cells in this disorder is analogous to that of the normal fetal ductal plate after 20 weeks gestation, suggesting that this developmental abnormality may take place during the latter half of gestation. CK7,8,18,19 staining is observed in the pathological state of the ductal plate cells [32]. The pathogenesis of ADPKD is closely related to keratin. A genetic disorder, ADPKD (1 in 1000) is a major contributor to end‐stage renal failure. Characterized by the emergence of multiple epithelial cysts, both renal and non‐renal, with extrarenal manifestations such as hypertension, heart valve issues, and brain aneurysms [33], it is caused by either PKD1 or PKD2 mutation [34]. It has been confirmed that there is a direct interaction between polycystic protein‐1 and keratin. Currently, IF protein vimentin is identified as a strong interacting partner of polycystic protein‐1. Cytokeratin K8, K18, and desmin have also been found to interact with P1CT, and these interactions are mediated by the crimp‐crimp motif in polycystic protein‐1 and IF proteins. Polycystic protein‐1 may use this association for structural, storage, or signaling functions. Polycystic protein‐1 also plays a role in the arrangement of keratin filaments. Mutations in PKD1 have an adverse impact on the aggregation of protein 17 (K17), and alterations in signal transduction resulting from K17 aggregates in the cytoplasm can disrupt the proper arrangement of the epidermal cytoskeleton, ultimately leading to cyst formation. Additionally, keratin 19 is localized exclusively within the epithelial cells of the cyst [35]. In normal kidneys, this protein is only visible in a few tubules. This feature may be useful for clinical identification of ADPKD [18].

### 3.3. Role of Keratin in Renal Tumors

Keratin is closely linked to tumor occurrence and is widely used in clinical tumor diagnosis and prognosis evaluation. In a study of cat tumors, researchers found that keratin and vimentin were co‐expressed in renal clear cell carcinoma (CRCC) and renal papillary carcinoma (PRCC) [36]. An immunohistochemical study of 42 renal cell tumor cases also revealed high keratin expression [37]. Besides, other studies have shown that keratin is important in classifying and subtyping renal clear cell and PRCC [38, 39]. Keratin can also aid the diagnosis of other kidney tumors, such as rare primary synovial sarcoma of the kidney [40] and undifferentiated and poorly differentiated tumors [41]. In addition, the keratin phenotype and distribution are different in different kidney tumors [42].

#### 3.3.1. K7 and Renal Tumors

Currently, K7 is the most researched keratin concerning renal cancer. In a feline renal cell carcinoma (RCC) study, the tumor cells K7 and K20 are positive except for cases of the solid type with clear cytoplasm (solid anaplastic), papillary type with columnar cells, and sarcomatoid types [43]. With the discovery of more and more key factors and immunohistochemical markers, the WHO classification of renal epithelial tumors is constantly changing. In four cases of adult renal epithelial tumors with unique morphology and immunophenotype, CK7 showed a diffuse strong immune response, and low cytokeratin CK19 and CK20 were completely negative [44]. Therefore, CK7 is essential for the classification of renal epithelial tumors. For the two most common subtypes of RCC, CRCC and PRCC, as well as RCCS with mixed forms of both, or RCCS containing papillary structures with clear cell lesions (CCC), CK7 is expressed and distributed differently in different tumors. It is helpful to classify these types of RCCS with vague forms [45]. In classifying RCC subtypes, some researchers have conducted a correlation analysis between radiomic features and CK7 to help accurate diagnosis [46]. Besides, CK7, together with p63, PAX8, and INI‐1, is the best immunohistochemical method for differentiating poorly differentiated urothelial cell carcinoma from high‐grade tumors in the renal collecting system [47]. In RCC associated with von Hippel–Lindau syndrome (VHL), CK7 stains are partially positive. This suggests that the immunophenotype of clear cells might be related to their differentiation status [48]. Besides, CK7 is associated with rare renal tumors. For example, in eosinophilic solid cystic renal cell carcinoma (ESCRCC) [49], renal thyroidoid follicular carcinoma (TLFCK) [49], low‐grade oncocytic tumor of the kidney (LOT) [50], clear cell papillary renal cell carcinoma (CCPRCC), primary renal pelvis lymphoepitheliomatoid carcinoma [51], the expression of CK7 was positive. Moreover, in small cell carcinoma of the renal pelvis and ureter, CK7 is rarely positive [52]. CK7 is also associated with benign tumors and benign nephropathy. In renal eosinophilic adenoma, a benign tumor, keratin 7 is one of the most widely used diagnostic staining methods, showing a diffuse positive response and staining of isolated tumor cells (< 5%) [53]. In metanephric adenoma (MA), the positive CK7 expression rate is about 20%, which is helpful for the diagnosis of MA when combined with other molecules [54]. There has been a debate regarding the differentiation between atrophic kidneys in benign nephropathy and thyroid‐like follicular kidney cancer. However, CK7 is almost not present in atrophic kidneys but is highly expressed in thyroid‐like follicular carcinoma. Consequently, CK7 can be a useful molecule in distinguishing between the two [55].

#### 3.3.2. Other Keratin and Renal Tumors

In Bellini tube carcinoma, a rare kidney tumor, we found strong positivity for keratin 5, 8, and 18, but no response for keratin 10 [56]. In addition to CK8 and CK18, a subpopulation of RCC also expresses CK19 positively [57]. Furthermore, the findings indicate that CK18 might have a significant impact on the advancement of RCC and could serve as a novel predictor of the disease. The expression of CK18 was linked to enhanced expression of snail, a transcription factor, and the expression of snail was positively associated with the progression of RCC in patients [58]. Cytokeratin 19 fragment CYFRA21‐1 is a widely used tumor marker in the clinic. In patients with renal urothelial carcinoma (UC), serum CYFRA21‐1 is elevated and can be used as a useful noninvasive indicator to evaluate treatment response [59]. However, when kidney function is reduced, if CYFRA is used as a marker of oral cancer, the content will be high, so kidney function will affect the expression of markers of other cancers [60].

#### 3.3.3. Possible Mechanisms of Keratin and Renal Tumorigenesis

Met is a proto‐oncogene product and a member of the tyrosine kinase growth factor receptor family with a ligand of HGF/SF. HGF/SF‐mediated activation of Met in mesenchymal cells may play a role in the transformation of cells into epithelial phenotypes. However, kidney cancers that co‐express cytokeratin and vimentin may originate from this cell transformation [61]. In addition, a study investigated the diagnostic and prognostic value of KRT19 methylation levels in RCC by evaluating the methylation and expression status of the promoter region of the KRT19 gene (the gene encoding CK19) in six renal cancer cell lines and 112 primary renal tumors [62]. The specific regulation in vivo is also affected by other genetic mechanisms, which need to be further studied.

## 4. How Does Keratin Regulate Kidney Disease

Keratin’s role in kidney disease will be elucidated further in this section, with its molecular mechanism.

### 4.1. Cell Death

Keratins act as multifaceted regulators of cell death: they can serve as shields against apoptosis, yet also drive non‐apoptotic demise through distinct pathways; abnormalities in their structure or function frequently act as triggers or accelerators of the dying process.

During apoptosis, K8/K18 keratins shield epithelial cells by weakening TNF‐family pro‐apoptotic signals such as TNF‐*α* and Fas. K8/K18 keratins first bind to the RNA‐binding protein HuR and compete for its interaction with the 3 ^′^ untranslated region (3 ^′^UTR) of death receptor 5 (DR5) mRNA, thereby repressing DR5 translation and reducing DR5 protein levels without changing DR5 mRNA abundance. The ensuing decrease in DR5 impairs assembly of the TRAIL‐induced death‐inducing signaling complex (DISC), so activation of caspase‐8 and cleavage of the BH3‐only protein Bid are diminished. Consequently, mitochondrial release of cytochrome c (cyto‐c) and loss of membrane potential are suppressed, ultimately lowering caspase‐3 activity and promoting cell survival [63]. In addition, researchers discovered that under basal stress, keratins K8/K18 act as a “phosphorylation decoy” by using residues such as Ser73 and Ser52 to dock p38/JNK kinases, thereby dampening pro‐apoptotic signals. Upon entry into apoptosis, caspase‐3 precisely cleaves K18 at Asp238 and Asp397, allowing keratin filaments to disassemble and reorganize so that programmed cell death proceeds in an orderly fashion. Mutation of these aspartates to non‐cleavable glutamates prevents keratin reorganization, leaves kinases persistently active, and forces the cell into necrosis [64].

Keratin can also be involved in programmed necrosis of cells, which is a non‐apoptotic mode of cell death. For example, in acidic microenvironments surrounding necrotic cells, surface‐exposed C‐terminal fragments of keratin K1/K10 are specifically bound by the dendritic‐cell endocytic receptor CD205 (cluster of differentiation 205, DEC205) in a pH‐dependent manner. This keratin‐mediated recognition triggers rapid internalization and subsequent antigen presentation by dendritic cells, establishing a phosphatidylserine‐independent “eat‐me” pathway that efficiently clears cells undergoing programmed necrosis [65]. Furthermore, our team has demonstrated that K20 mediated its protective effect against AKI through the inhibition of ferroptosis in renal tubular epithelial cells by regulating the exosomal secretion of Peroxiredoxin 2(PMID: 40711819).

In some cases, abnormalities in keratin may trigger or speed up the process of cell death. For example, deletion or mutation of the keratin gene can lead to decreased cellular mechanical stability, hemifibrous instability, and increased cell migration and invasion capacity. Alterations in keratin expression profiles in cancer can be linked to the aggressiveness and ability to spread tumors, including the reduction of K8/18, which enhances the collective movement and aggressiveness of specific epithelial cancer cells [66].

Keratin’s intricate and multifarious roles offer essential insights into cell death processes and the development of treatments for cell death‐related illnesses. Nevertheless, the precise regulatory mechanisms of keratin in cell death and the resemblances and disparities of keratin action in various kinds of cells are still essential areas for further exploration. In conclusion, keratin plays a protective part in cell death and is implicated in controlling cell death pathways.

### 4.2. Autophagy

The role of keratin in autophagy is mainly related to the stability of cell structure and the dynamic changes of intracellular membranes. Although keratin itself is not usually involved in autophagosome formation or protein degradation, its role in cytoskeleton maintenance and cell response to external stress can indirectly influence the occurrence of autophagy.

Keratin, a vital element of the cytoskeleton, aids in sustaining cell form and mechanical steadiness, particularly when cells are reacting to stress, such as inadequate nutrition. This stabilizing of the cytoskeleton helps avert excessive depletion or harm to cells during autophagy [67]. Furthermore, keratin is implicated in cellular stress reactions such as DNA damage, oxidative strain, or alterations in microenvironmental conditions—these can also prompt autophagy as a means of clearing out damaged proteins and organelles [68]. For example, studies have shown that in renal tubular epithelial cells, starvation or ischemia induces p38 mitogen‐activated protein kinase (p38 MAPK)‐mediated phosphorylation of keratins K8/K18. This phosphorylation causes keratin filaments to disassemble and form bridges with microtubule‐associated protein 1 light chain 3 (LC3), physically tethering the isolation membrane to lysosomes and thereby ensuring efficient autophagosome–lysosome fusion. As a result, damaged mitochondria are removed, reactive oxygen species (ROS) are suppressed, and the survival rate of tubular cells is ultimately increased [69].

As mentioned in the previous section, keratin also plays a role in apoptosis, which has a complex relationship with autophagy. In some cases, abnormalities in keratin may lead to changes in the way cells die, which may also indirectly affect the regulation of autophagy.

Although keratin is not a direct builder of autophagosomes, it plays an indirect role in supporting the formation of isolation membranes and autophagosomes by providing a stable cellular environment. Further study of the interaction between keratin and autophagy will help us better understand how cells maintain homeostasis in response to external stress.

### 4.3. Endoplasmic Reticulum (ER) Stress

Under the electron microscope, the peripheral ER showed nanoscale proximity to keratin filaments and pontoplasm plaques, and keratin disturbance affected the expression of ER stress transcripts [70]. ER stress is the way to eliminate unfolded and misfolded proteins. Studies show that in the glomerulus, when podocytes lack keratins K8/K18/K19, the ER loses its cytoskeletal scaffold. This causes the unfolded protein response sensor PERK (protein kinase R‐like ER kinase) to remain active, rapidly elevating levels of the transcription factor ATF4 (activating transcription factor 4) and the pro‐apoptotic protein CHOP (C/EBP homologous protein), leading to massive podocyte death and glomerular sclerosis [71]. Tracing the molecular mechanism, when TRIM13 (tripartite motif‐containing protein 13) expression falls due to promoter hypermethylation, ubiquitin‐mediated degradation of K18 cannot be blocked, likewise enhancing ER stress and increasing collagen IV secretion by mesangial cells, thereby worsening glomerular sclerosis [72].

Meanwhile, in tubular injury the same interplay is observed: ischemia–reperfusion causes caspase‐3 to cleave K18, the keratin network collapses, the ER chaperone BiP (immunoglobulin heavy‐chain binding protein) is up‐regulated, and both IRE1 (inositol‐requiring enzyme 1) and PERK pathways are amplified, forcing renal tubular epithelial cells into apoptosis and driving acute damage toward chronic fibrosis [73, 74].

On the other hand, studies focused on diabetic kidney disease demonstrate that maintaining the keratin scaffold markedly suppresses ER stress. Under high‐glucose conditions K8 in glomerular endothelial cells is degraded, calcium leakage keeps IRE1‐XBP1 (X‐box binding protein 1) continuously active, and inflammatory cytokines IL‐6 (interleukin‐6) and MCP‐1 (monocyte chemoattractant protein‐1) were massively released [71, 75]; if an Src protein inhibitor is used to block K8 tyrosine phosphorylation, keratin filaments re‐anchor to the ER, IRE1*α* activity declines, and expressions of CHOP and fibronectin are reduced, thereby alleviating diabetic renal injury [76].

Collectively, these findings indicate that the integrity of the keratin K8/K18/K19 network—by mechanically tethering the ER and preserving ER Ca^2+^ homeostasis—determines the intensity and duration of ER stress. Loss or cleavage of these keratins amplifies ER stress, accelerates apoptosis and fibrosis in either glomerular or tubular cells, and positions the “keratin‐ER stress” axis as a common and druggable node for progression of various kidney diseases.

### 4.4. Post‐Transcriptional Modification

It has been suggested that there are interindividual differences in the phenotype of keratins, possibly related to post‐transcriptional modifications [77]. There are few studies on the post‐transcriptional modification of keratin. Djudjaj et al. were the first to demonstrate that after renal ischemia, keratin K8 Ser73 and K18 Ser52 are rapidly phosphorylated by p38 mitogen‐activated protein kinase (p38 MAPK) and c‐Jun N‐terminal kinase (JNK), forming a “hyper‐phosphorylated keratin band.” This hyper‐phosphorylated state causes keratin filaments to disassemble into soluble tetramers that act as a “phosphate‐protein sponge,” adsorbing excess stress kinases and preventing their diffusion to mitochondria and death receptor platforms, thereby attenuating apoptosis in renal tubular epithelial cells. This finding provided the first direct evidence that “hyper‐phosphorylation of keratins after kidney injury” possesses a biological function and laid the foundation for future research [78].

For other keratins, K17 has two phosphorylation sites, threonine 9 (Thr9) and serine 44 (Ser44) are identified, and K17 phosphorylation is increased undergrowth and stress conditions [79]. K1 and K10 have been reported to be substrates of peptidyl arginine deaminases (PADs), which catalyze derefinement (or citlination), a post‐transcriptional modification [80].

## 5. Summary and Future Directions for Development

Over time, the scientific community has increased its attention to the relationship between keratin and kidney function. The type, expression, and mutation of keratin can vary depending on the physiological or pathological state of the kidney. Among them, the function of K8, K18, and K19 in the kidney is gradually clear, and the potential role of other keratins is also constantly discovered. In kidney disease such as AKI, chronic disease, and kidney tumors, different subtypes of keratin are associated with the disease in different ways. In pathological diagnosis, as one of the immunohistochemical molecules, keratin plays an important role in the classification of renal lesions [81]. Therefore, keratin is often used as a biomarker in serological detection. Antibodies to various subtypes of keratin are also increasing, and more animal sources of monoclonal antibodies, such as mice and rabbits, are being sought [82].

Compared with the currently available biomarkers for kidney diseases, what advantages will the detection of keratin bring? Traditional indicators such as creatinine are susceptible to muscle mass and physiological status, which easily leads to missed diagnosis of early renal injury in elderly and patients with sarcopenia. As a tubular epithelial cell‐specific biomarker, keratin can realize ultra‐early diagnosis of subtle tubular damage and accurately identify drug‐induced and contrast‐induced AKI at the initial stage when albumin and creatinine remain normal [15]. In terms of prognosis, persistent elevation of urinary keratin effectively predicts the long‐term risk of CKD deterioration and ESRD occurrence, acting as an independent risk factor for adverse renal outcomes [83]. Clinically, noninvasive urinary keratin testing can effectively reduce the reliance on repeated renal biopsy and is applicable for long‐term follow‐up of all‐age kidney disease patients, including pregnant women and children [84, 85].

In which aspects do we still need to make efforts? Firstly, it is necessary to broaden the research on kidney diseases and the types of keratins. We still lack more data on keratin in kidney diseases, such as kidney transplantation and benign kidney tumors. In addition, we should not be limited to keratin proteins such as K8 and K18 that have been studied extensively. We should also explore the expression levels and functions of other keratin proteins. Secondly, the mechanism is still less studied and unclear. Although some studies have mentioned that keratin can regulate kidney diseases through different pathways, such as cell death, autophagy, ER stress, post‐transcriptional modification, etc. However, the specific path is still unclear, which requires more experiments. Further exploration is needed to determine how other factors affect keratin in various kidney diseases. Thirdly, few targeted drugs exist. Future research should aim to determine its clinical value, rather than being limited to the effect of biomarkers.

In conclusion, by summarizing the relationship between keratin and the kidney, this review hopes to draw researchers’ attention to the potential fields that are worthy of further exploration and expansion.

## Author Contributions

**Shitian Che:** writing – original draft, writing – review and editing, conceptualization; **Xiaolu Huang:** writing – original draft, writing – review and editing; **Shuxiang Yan:** writing – review and editing; **Baiyu Feng:** writing – review and editing; **Lijun Yin:** supervision, conceptualization, writing – review and editing; **Anqun Chen:** supervision, funding acquisition, conceptualization, writing – review and editing. All authors agree to be accountable for all aspects of the work. Shitian Che, and Xiaolu Huang contributed equally to this work and should be considered co‐first authors.

## Funding

This study was supported by National Natural Science Foundation of China, 10.13039/501100001809, NO. 82222013, 82470731; Natural Science Foundation of Hunan Province, 10.13039/501100004735, No. 2025JJ30048; Degree & Postgraduate Education Reform Project of Central South University, 2025JGB014; Degree and Graduate Education Reform in Hunan Province, 2025JGYB031; National Key Clinical Specialty Scientific Research Project of the Second Xiangya Hospital, Central South University.

## Disclosure

All authors read and approved the final manuscript.

## Ethics Statement

The authors have nothing to report.

## Consent

The authors have nothing to report.

## Conflicts of Interest

The authors declare no conflicts of interest.

## Data Availability

The datasets generated and/or analyzed during the current study are available from the corresponding author on reasonable request.
